# Ectopic hTERT expression facilitates reprograming of fibroblasts derived from patients with Werner syndrome as a WS cellular model

**DOI:** 10.1038/s41419-018-0948-4

**Published:** 2018-09-11

**Authors:** Shuyan Wang, Zhongfeng Liu, Yanxia Ye, Bingnan Li, Tiantian Liu, Weiqi Zhang, Guang-Hui Liu, Y. Alex Zhang, Jing Qu, Dawei Xu, Zhiguo Chen

**Affiliations:** 1 Cell Therapy Center, Xuanwu Hospital, Capital Medical University, and Key Laboratory of Neurodegeneration, Ministry of Education, Beijing, China; 2 Center of Neural Injury and Repair, Beijing Institute for Brain Disorders, Beijing, China; 3 State Key Laboratory of Stem Cell and Reproductive Biology, Institute of Zoology, Chinese Academy of Sciences, Beijing, China; 4 Division of Hematology, Department of Medicine and Center for Molecular Medicine, Karolinska Institutet and Karolinska University Hospital, Stockholm, Sweden; 5 Department of Pathology, Shandong University School of Medicine, Jinan, China; 6 National Laboratory of Biomacromolecules, CAS Center for Excellence in Biomacromolecules, Institute of Biophysics, Chinese Academy of Sciences, Beijing, China

## Abstract

The induced pluripotent stem cell (iPSC) technology has provided a unique opportunity to develop disease-specific models and personalized treatment for genetic disorders, and is well suitable for the study of Werner syndrome (WS), an autosomal recessive disease with adult onset of premature aging caused by mutations in the *RecQ like helicase (WRN)* gene. WS-derived fibroblasts were previously shown to be able to generate iPSCs; however, it remains elusive how WS-derived iPSCs behave and whether they are able to mimic the disease-specific phenotype. The present study was designed to address these issues. Unexpectedly, we found that a specific WS fibroblast line of homozygous truncation mutation was difficult to be reprogrammed by using the Yamanaka factors even under hypoxic conditions due to their defect in induction of hTERT, the catalytic unit of telomerase. Ectopic expression of hTERT restores the ability of this WS fibroblast line to form iPSCs, although with a low efficiency. To examine the phenotype of WRN-deficient pluripotent stem cells, we also generated WRN knockout human embryonic stem (ES) cells by using the CRISPR/Cas9 method. The iPSCs derived from WS-hTERT cells and WRN-/- ESCs are fully pluripotent, express pluripotent markers and can differentiate into three germ layer cells; however, WS-iPSCs and WRN-/- ESCs show S phase defect in cell cycle progression. Moreover, WS-iPSCs and WRN-/- ESCs, like WS patient-derived fibroblasts, remain hypersensitive to topoisomerase inhibitors. Collectively, WS-derived iPSCs and WRN-/- ESCs mimic the intrinsic disease phenotype, which may serve as a suitable disease model, whereas not be good for a therapeutic purpose without gene correction.

## Introduction

Werner syndrome (WS) is an autosomal recessive syndrome characterized by the onset of premature aging and age-related disorders in early adulthood, and results predominantly from loss-of-function mutations in the *WRN* gene encoding the RecQ helicase^1–4^.

Induced pluripotent stem cells (iPSCs) have shown great potential for applications in modeling the disease pathogenesis, screening for novel drug compounds, and developing new therapies^4–7^. Given the great advantage of the iPSC technology in capturing phenotypes of genetic diseases, two groups have recently tested the generation of iPSCs derived from WS patient fibroblasts^8,9^. Despite this, it remains elusive how WS-derived iPSCs behave and whether they are able to mimic the disease-specific phenotype. In addition, WS is attributable to loss-of-function mutations in the *WRN* gene, but accelerated telomere shortening is widespread and significantly contributes to pathological alterations in WS patients^10–12^. Therefore, the comprehensive dissection of the relationship between hTERT or telomere dynamics and the generation/proliferation of iPSCs from WS cells should gain better insights into the iPSC WS model for mechanistic studies and personalized cell therapy. Here, we sought to address these questions by defining how exactly hTERT affects generation, phenotype maintenance and other properties of iPSCs from WS fibroblasts.

## Results

### The Yamanaka factors fail to stimulate iPSC generation from one specific WS-derived fibroblast line

Fibroblasts used in this study included three WS patients-derived fibroblast lines (The genetic alterations detailed in Methods), AG03141 (homozygous 2476C > T mutation in the *WRN* gene), AG00780 (homozygous 1336C > T mutation in the *RECQL2* gene), and AG06300 (with the polymorphism—a leucine for phenylalanine replacement at amino acid 1074 of the WRN protein). In addition, we used two human ES cell lines deficient in WRN. The first line WRN-ES1 has been published in a previous report^13^, and the second line WRN-ES2 was generated using the CRISPR/Cas9-mediated knockout method (Supplementary Fig. S1). WRN-ES1 and WRN-ES2 were derived from an iso-control H9 ES cell line. The three ES cell lines were differentiated to human mesenchymal stromal cells (hMSCs), and flow cytometry-purified as CD73/CD90/CD105 triple-positive hMSC population. The purified hMSCs were also used as starter cells for induction of iPSCs.

**Fig. 1:**
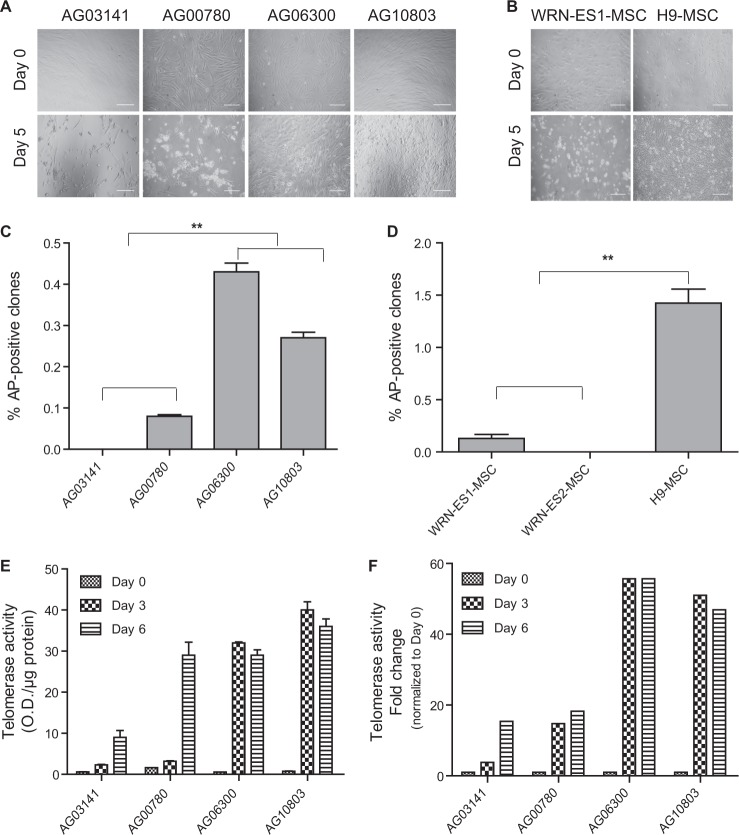
The Yamanaka factors fail to stimulate iPSC generation from one specific WS fibroblast line. **a** Representative images of WS (AG03141, AG00780, AG06300) and WT (AG10803) fibroblasts before Sendai virus infection and on Day 5 post-infection. Bar = 100 μm. **b** Representative images of WRN knockout (WRN-ES1) and WT (H9) human embryonic stem cells-derived MSCs before Sendai virus infection and on Day 5 post-infection. Bar = 100 μm. **c** The efficiency of iPSC generation from WS and WT fibroblasts. Values represent the mean percentage of alkaline phosphatase (AP)-positive clones among the number of plated cells. **P < 0.001. **d** The efficiency of iPSC generation from WRN-ES1-, WRN-ES2-, and H9-derived MSCs. Values represent the mean percentage of alkaline phosphatase (AP)-positive clones among the number of plated cells. **P < 0.001. **e** Telomerase activity of WS and WT groups at reprogramming Day 0, 3, and 6. **f** Fold change of telomerase activity at different reprogramming time points (Day 3, Day 6) normalized to that of Day 0 in WS and WT groups. WS Werner syndrome, WT Wild-type, WRN-ES WRN knockout human embryonic stem cells, H9 WT human embryonic stem cells

In an attempt to reprogram the above fibroblasts and passage number 10 (p10) hMSCs (including WS and WT cells) to iPSCs, Sendai virus encoding the Yamanaka factors (Oct-4, Sox2, Klf4, C-Myc) were added. WS and WT cells were plated at the same density prior to viral infection. On the 3rd day post-infection, WS cells of homozygous truncation genotype (AG03141, AG00780, and WRN-ES-MSCs) started to exhibit a senescent phenotype. On the 5th day, the numbers of surviving cells in the WS truncation mutation groups were significantly lower than those in the WT (AG10803 and H9-MSCs) groups (Fig. 1a, b). After replating on MEF feeder cells, some cell lines started to show alkaline phosphatase (AP)-positive iPSC clones around 3 weeks post-infection (Fig. 1c, d). In general, iPSC induction rates were lower with cells of homozygous truncation mutation genotype than those of missense mutation and WT genotype. Particularly, no iPSC clones were detected from AG03141 fibroblasts and p10 WRN-ES2-MSCs. In total, four dishes with 4 × 10^5^ AG03141 WS fibroblasts were tested for iPSC derivation and observed for 28 days following replating on MEFs but no iPSC clones appeared. The tests on AG03141 fibroblasts were repeated twice with identical results. Because Batista et al.^14^ previously showed the improved reprogramming efficiency of dyskeratosis congenital (DKC) cells with telomere dysfunction by using a low oxygen concentration (5%), we tried this same strategy, but failed to observe the formation of any iPSC clones from AG03141 WS cells.

Aging and cellular senescence may increase the barrier to iPSC reprogramming. We thus compared hMSCs of early and late passages for the induction rates (Supplementary Fig. S2A, B). hMSCs of p10 showed signs of senescence as evidenced by β-gal staining, which was not seen in those of early passages (p5) (Supplementary Fig. S2C). The reprogramming efficiencies to iPSCs were lower for p10 hMSCs vs. p5 hMSCs (Supplementary Fig. S2D).

### Defective telomerase activation occurs during reprograming of AG03141 WS fibroblasts and ectopic hTERT expression bypasses the barrier to iPSC induction

Robust telomerase/hTERT activation occurs in and is required for reprogramming while telomerase deficiency impaired the efficiency of iPSC generation^8,14,15^. Thus, we determined whether the inability of WS cells to be reprogrammed was due to defective hTERT induction or telomerase activation. As expected, both WS and WT fibroblasts showed negligible levels of telomerase activity. On Day 3, reprogramming process robustly upregulated telomerase activity in WT fibroblasts and missense mutation AG06300 WS fibroblasts, while only limited increase occurred in WS cells of truncation mutation genotypes (Fig. 1e, f).

**Fig. 2:**
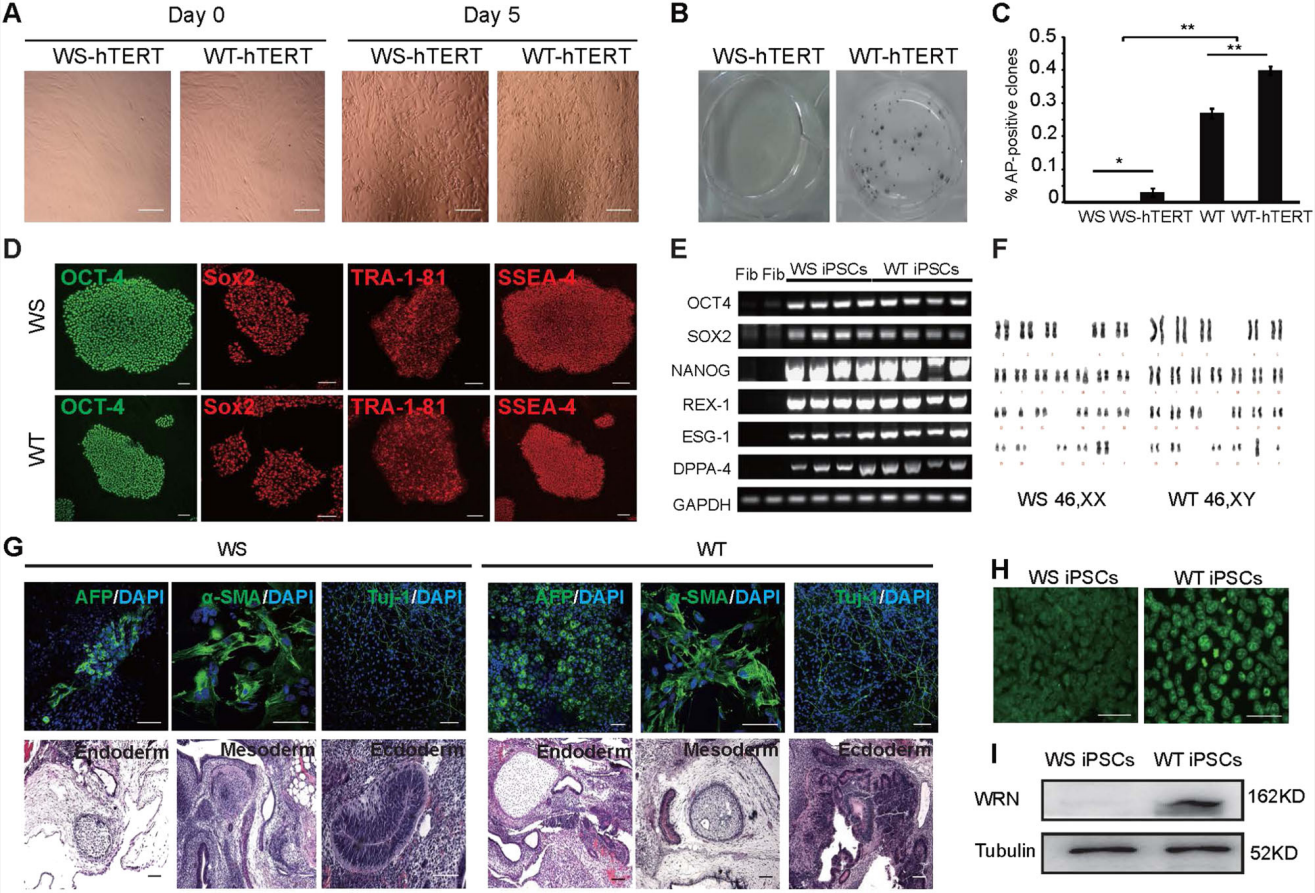
Defective telomerase activation occurs during reprograming of WS fibroblasts. **a** Representative images of WS-hTERT (AG03141) and WT-hTERT (AG10803) fibroblasts before Sendai virus infection (Day 0) and on Day 5 post-infection. Bar = 100 μm. **b** AP staining results of WS-hTERT and WT-hTERT groups on Day 21. **c** The efficiencies of iPSC generation from WS (AG03141), WS-hTERT (AG03141-hTERT), WT (AG10803), and WT-hTERT (AG10803-hTERT) fibroblasts. Values represent the mean percentage of alkaline phosphatase (AP)-positive clones among the number of plated cells. **P < 0.001. **d** Immunofluorescent staining for OCT4, Sox2, TRA-1-81, and SSEA-4 on WS and WT iPSCs. **e** RT-PCR results of gene expression. Fib: fibroblasts. **f** Karyotype of WS and WT iPSCs. **g** Differentiation of WS and WT iPSCs to cells of the three germ layers by forming embryoid bodies in vitro and teratoma formation in vivo. **h** Staining for WRN protein on WS and WT iPSCs. **i** Western blotting analysis for WRN protein expression in WS and WT iPSCs

Given the observation that insufficient increase of telomerase activity was associated with failure of iPSC induction, we introduced hTERT (Supplementary Fig. S3) into AG03141 WS and AG10803 WT fibroblasts prior to reprogramming. Interestingly, with hTERT expression, AG03141 WS-hTERT fibroblasts exhibited a better survival on the 5th day post-infection (Fig. 2a), and AP-positive iPSC clones did show up from those AG03141 WS-hTERT cells (Fig. 2b, c), despite at a much lower efficiency compared with WT and WT-hTERT groups (Fig. 2c). Also, we observed a significantly increased number of iPSC clones formed from WT-hTERT cells than from WT fibroblasts without ectopic hTERT (Fig. 2c). Both WS-hTERT and WT-hTERT iPSCs expressed pluripotent markers Oct-4, Sox2, TRA-1-81 and SSEA-4 (Fig. 2d). Reverse transcription PCR (RT-PCR) results proved that both WS and WT iPSCs expressed pluripotent markers OCT-4, SOX2, NANOG, REX-1, ESG1, and DPPA4 (Fig. 2e). WS-hTERT and WT-hTERT iPSCs exhibited normal karyotype (Fig. 2f) and could both differentiate to the three germ layer cells including endoderm (AFP-positive), mesoderm (α-SMA-positive), and ectoderm (Tuj-1-positive) cells (Fig. 2g upper panel). When injected into immunodeficient SCID mice, WS- and WT-iPSCs could form teratoma consisting of tissues of the three germ layers (Fig. 2g lower panel). Taken together, telomerase activation enables AG03141 WS fibroblasts to undergo a successful reprogramming. As expected, AG03141 WS-iPSCs remained negative for WRN expression, as shown by immunofluorescent staining (Fig. 2h) and western blotting (Fig. 2i).

**Fig. 3:**
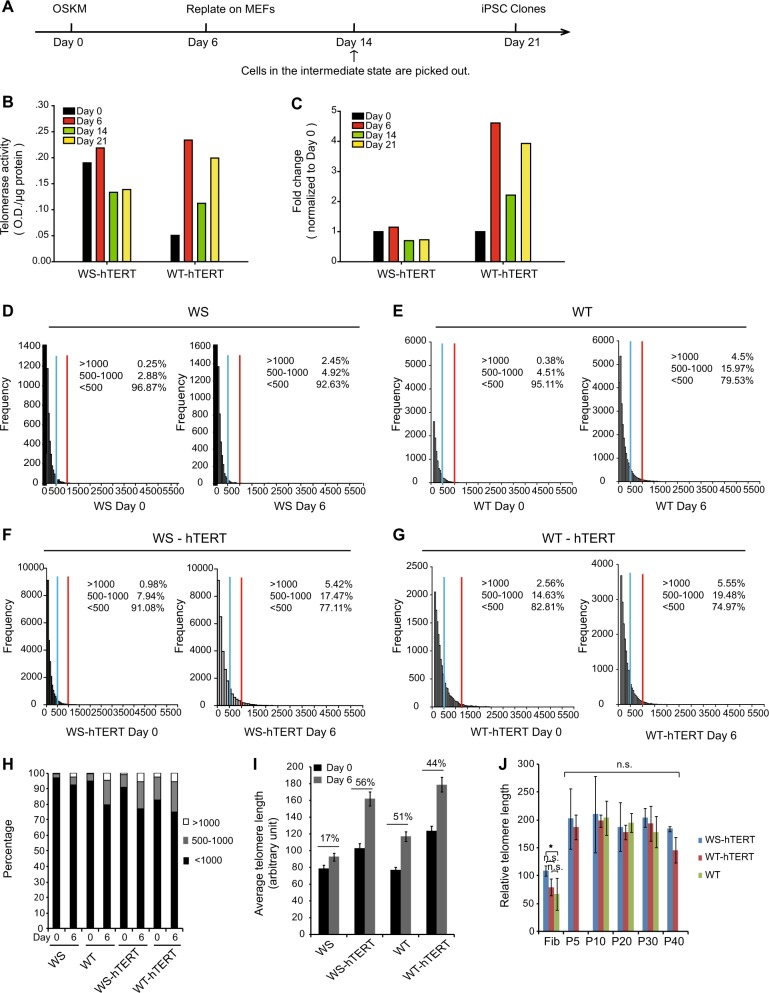
Ectopic hTERT expression bypasses their barrier to iPSC induction. **a** Scheme of iPSC generation and analysis at various time points. **b** Telomerase activity of AG03141 WS-hTERT and AG10803 WT-hTERT groups at reprogramming Day 0, 6, 14, and 21. **c** Fold change of telomerase activity at different reprogramming time points (Day 6, Day 14, and Day 21) normalized to that of Day 0 in AG03141 WS and AG10803 WT groups. **d**–**g** Analysis of telomere length on Day 0 and Day 6 of reprogramming. **h** Distribution of cells with various range of telomere length ( < 500, 500–1000, and > 1000). **i** The average telomere length on reprogramming Day 0 and Day 6. **j** Relative telomere length of iPSCs at different passage numbers measured by using Flow-FISH. The values represent the mean fluorescence intensity of telomere DNA sequence specific probes. In all, 4 × 10^4^ iPSC cells were analyzed in each run. The experiments were repeated four times. (WS-hTERT iPSCs were generated from AG03141-hTERT fibroblasts; WT-hTERT iPSCs were generated from AG10803-hTERT fibroblasts). Bar = 100 μm; WS Werner syndrome, WT Wild-type

At different time points (Fig. 3a, Day 0, Day 6, Day 14, and Day 21) along the course of reprogramming, dynamics in telomerase activity was further measured (Fig. 3a–c, and Supplementary Fig. S4). Telomerase activity was constitutively maintained at high levels in AG03141 WS-hTERT and AG10803 WT-hTERT fibroblasts during the observed period.

To examine whether hTERT expression lengthens telomere, we measured telomere length (TL) of cells prior to and 6 days after virus infection (Fig. 3a) using Q-FISH. On Day 0 (prior to infection), 0.25% of WS fibroblasts showed a TL longer than 1000 arbitrary units (arbitrary fluorescence intensity, relative index of TL), and 2.88% of WS cells between 500 and 1000, lower than those in WT fibroblasts (0.38%, > 1000; 4.51%, 500–1000, respectively, Fig. 3d, e). Stable expression of hTERT brought up the proportion of fibroblasts with longer telomeres in both WS group (0.98%, > 1000; 7.94%, 500–1000; Fig. 3f, h) and WT group (2.56%, > 1000; 14.63%, 500–1000; Fig. 3g, h). The average TL among the total cell population was also enhanced by hTERT expression (WS, from 79 to 92.4; WS-hTERT, from 103.2 to 161.9; WT, from 76.8 to 116.6; WT-hTERT, from 123.5 to 179; Fig. 3i).

Six days of the reprogramming process also increased the percentage of cells with longer telomeres (Fig. 3d–g). Nevertheless, on Day 6, the percentages of cells with longer telomeres in WS cells (2.45%, > 1000; 4.92%, 500–1000; Fig. 3d) were lower than those of the other three groups (WT: 4.5%, > 1000; 15.97%, 500–1000; WS-hTERT: 5.42%, > 1000; 17.47%, 500–1000; WT-hTERT: 5.55%, > 1000; 19.48%, 500–1000; Fig. 3e–g). The average TL was extended by the reprogramming process on Day 6 by 17%, 56%, 51%, and 44% in WS, WS-hTERT, WT, and WT-hTERT group, respectively (Fig. 3i).

To test whether telomere continue to be elongated with iPSC division, we measured TL at different passage numbers (P5, P10, P20, P30, and P40) in WS-hTERT, WT-hTERT, and WT iPSCs using Flow-FISH (Fig. 3j). Despite the difference in the baseline TL in the fibroblast lines, telomere was elongated to similar levels in the iPSCs at passage 5, and TL was stable along iPSC passage up to passage 40 (Fig. 3j).

### Constitutive hTERT expression is required for cell cycle progression of WS cell-derived iPSCs

The above data showed that AG03141 WS-hTERT iPSCs had pluripotent property, expressed pluripotent markers and differentiation ability comparable with AG10803 WT-iPSCs or WT-hTERT iPSCs. However, it is possible that the exogenous expression of hTERT may have masked defects caused by the *WRN* mutation. Using inducible hTERT (ihTERT) iPSCs and WRN knockout ES cells may help answer this question. Therefore, we adopted a doxycycline (Dox)-inducible system to generate iPSCs. The fibroblasts (AG03141 and AG10803) were infected with Dox-inducible hTERT lentiviral vectors (Supplementary Fig. S5) and selected with puromycin. With the addition of Dox, WS-ihTERT and WT-ihTERT fibroblasts were converted to iPSCs.

**Fig. 4:**
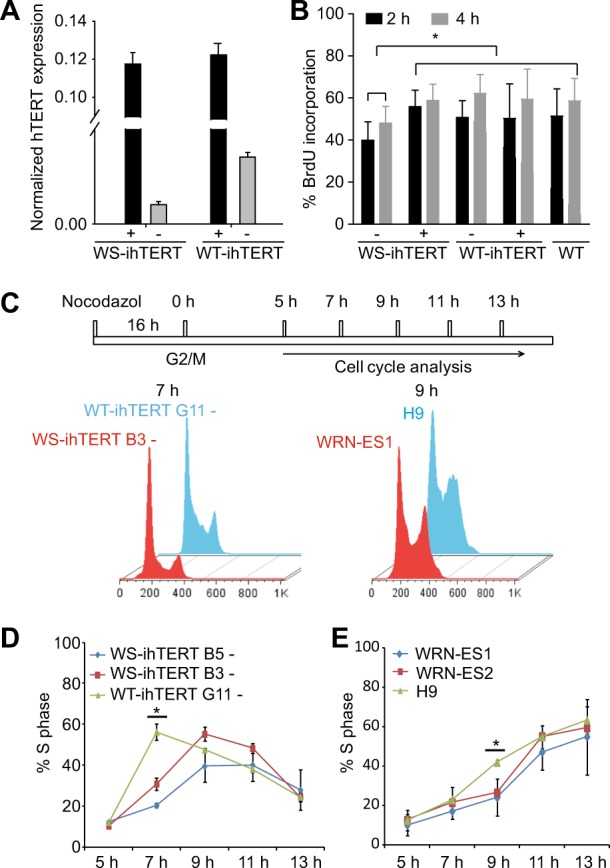
Constitutive hTERT expression is required for cell cycle progression of WS cell-derived iPSCs. **a** hTERT expression in WS-ihTERT and WT-ihTERT iPSCs at passage 10 (P10). **b** BrdU incorporation in WS and WT iPSCs with inducible hTERT with or without Dox. **c** Cell cycle analysis at different time points after synchronization with nocodozal. **d**, **e** Percentages of cells at S phase after synchronization with nocodozal. WS-ihTERT-, Werner syndrome iPSCs (AG03141) with inducible hTERT without Dox treatment; WT-ihTERT-, Wild-type iPSCs (AG10803) with inducible hTERT without Dox treatment

When Dox was removed from cultured WT- and WS-ihTERT iPSCs at passage 10, remaining endogenous hTERT expression was significantly low (Fig. 4a). Of note, WS-ihTERT iPSCs expressed even lower endogenous hTERT mRNA, less than 1/3 of that in WT-ihTERT iPSCs (Fig. 4a). Without Dox, WS-ihTERT iPSCs showed a lower attachment efficiency and longer population doubling time compared with WT-ihTERT. WT-ihTERT iPSCs were normally passaged at a ratio of 1:6 and became confluent in 4–5 days, in contrast to WS-hiTERT that had to be passaged at a ratio of 1:3–4 with a mean doubling time of 7 days. To further examine the proliferation capacity, BrdU was pulsed for 2 or 4 h and the percentages of BrdU-positive cells were counted (Fig. 4b). Regardless of Dox addition, WT-ihTERT iPSCs showed comparable rates of BrdU incorporation, which was similar to those of WT iPSCs (derived from AG10803 WT fibroblasts without ectopic hTERT expression) and of Dox-treated WS-ihTERT iPSCs. However, the BrdU incorporation rate was significantly lower in WS-ihTERT in the absence of Dox, compared with other groups (Fig. 4b). We then looked into the cell cycle progression of two AG03141 WS-ihTERT iPSC clones (clones B3 and B5) and the AG10803 WT-ihTERT control. Cells were treated with nocodazole for 16 h to be synchronized at G2/M stage (Supplementary Fig. S6), and then switched to normal iPSC medium for 5 h (Supplementary Fig. S6), followed by flow cytometry analysis at different time points (Fig. 4c). In all, 7 h after switching to normal iPSC medium, 56.3 ± 4.0% of WT iPSCs entered S phase whereas only 20.4 ± 1.0% and 30.0 ± 2.9% of the two WS iPSC clones did so, suggesting that WS-iPSCs proceed to S phase at a slower pace (Fig. 4d). In addition to iPSCs, we also looked into two WRN-/- ES cells (WRN-ES1 and WRN-ES2) and the iso-control H9 ES cells. Compared to the three iPSC lines, the three ES lines generally took longer time to complete a cell cycle. Yet at 9 h, similarly, there was a higher proportion of cells at S phase in wild-type H9 ES cells vs. WRN-/- ES lines (Fig. 4e).

### WS-iPSCs are more sensitive to treatment of anticancer drug Camptothecin

**Fig. 5:**
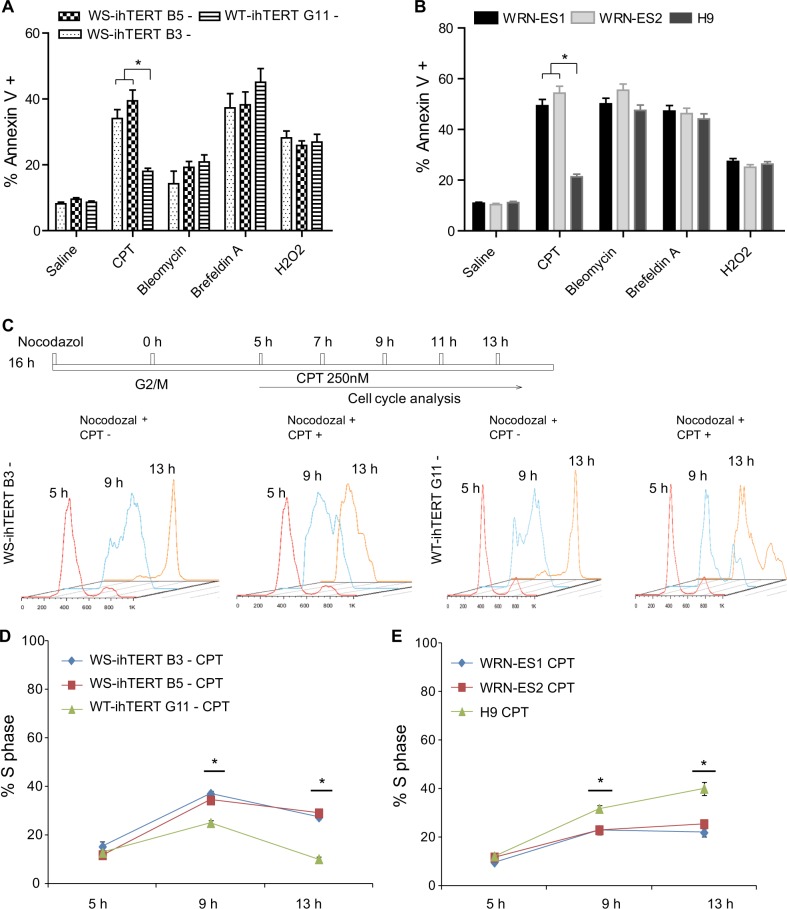
WS-iPSCs and WRN-ES cells are more sensitive to treatment of anticancer drug Camptothecin. **a** Percentages of apoptotic iPSCs treated with Camptothecin (CPT), Bleomycin, Brefeldin A, or H_2_O_2_. **b** Percentages of apoptotic ES cells treated with CPT, Bleomycin, Brefeldin A, or H_2_O_2._ * *P* < 0.005. **c** Cell cycle analysis after synchronization with nocodozal in the presence or absence of CPT. **d**, **e** Percentages of cells in S phase with CPT treatment at different time points (5, 9, and 13 h)

We next investigated how WS- and WT-iPSCs reacted to different stressors. Four kinds of stressors were applied to the cells, Camptothecin (CPT), Bleomycin, Brefeldin A, and hydrogen peroxide (H_2_O_2_). Low doses (25–100 nM) of CPT induce replication fork slowing and reversal without leading to detectable levels of double stranded DNA breaks (DSBs), and at higher concentrations ( > 100 nM) CPT induces DNA breaks^16,17^. iPSCs and ESCs of different groups (AG03141 WS-ihTERT + , WS-ihTERT-, AG10803 WT-ihTERT + , WT-ihTERT-, WT, WRN-ES1, WRN-ES2, and H9) were subjected to the treatment of individual stressors and the proportion of apoptotic (Annexin V + ) cells measured by using flow cytometry. The saline group did not receive treatment of any of the four stressors, and the dissociation process produced the baseline apoptotic levels. Among the four stressors, only CPT showed differential effect on the two AG03141 WS iPSC clones vs. AG10803 WT iPSCs (Fig. 5a without Dox, and Supplementary Fig. S7A with Dox). Following CPT treatment, around 43% WS-ihTERT + (with Dox) and 33–39% WS-ihTERT- (without Dox) iPSCs were positive for Annexin V, significantly higher than those of the other groups (14.7 ± 2.5% for WT-ihTERT-G11 + , 18.0 ± 2.1% for WT-ihTERT-G11-, and 10.2 ± 0.7% for WT iPSCs). Similar phenomenon was observed as to the three ES cell lines. Only CPT treatment showed differential effect on the induced apoptosis of WRN-ES1 and WRN-ES2 vs. H9 iso-control ES lines (Fig. 5b). We further tested different concentrations of CPT on induction of apoptosis of WRN-ES1 vs. H9 ES cells (Supplementary Fig. S7B). CPT doses of 250 nM and higher showed a differential effect on the apoptosis levels. We, therefore, chose 250 nM CPT in the subsequent experiments, and Fig. S7C represented the flow cytometry data of apoptosis tests in AG03141 WS-ihTERT (Dox-) and AG10803 WT-ihTERT (Dox-) cells following saline or 250 nM CPT treatment. Additional doses of bleomycin and brefeldin A were tested on AG03141 WS-ihTERT and AG10803 WT-ihTERT with or without Dox, and no differential effect of the drugs was observed (Supplementary Fig. S7D, E).

The above data showed that loss-of function of WRN rendered the iPSCs more sensitive to CPT-induced apoptosis. We then asked at which stage of cell cycle WRN plays a role in this context. WS and WT iPSCs/ESCs were synchronized with nocodozal and changed back to normal iPSC medium for 5 h, followed by cell cycle analysis with or without CPT treatment (Fig. 5c, represented by iPSCs). At 13 h after removal of nocodozal, CPT (250 nM) treatment rendered the two WS-ihTERT clones (Dox-) stuck at S phase, and failed to proceed to the next phase of cell cycle. In contrast, CPT-treated WT-ihTERT (Dox-) were still able to proceed to the next phase (Fig. 5d). The ES lines used in the study had an intrinsically different temporal rhythm of cell cycle than the iPSC lines. It took a longer period of time for the ES lines to complete a full cell cycle. Despite this, the differential effect of CPT treatment on ES cells of wild-type vs. WRN-/- genotypes was similar. WRN-ES1 and WRN-ES2 cells were halted at S phase by CPT treatment, but wild-type H9 iso-control ES cells were not (Fig. 5e), suggesting that sensitivity to CPT treatment may be a common phenotype of WRN-deficient iPS/ES cells.

**Fig. 6:**
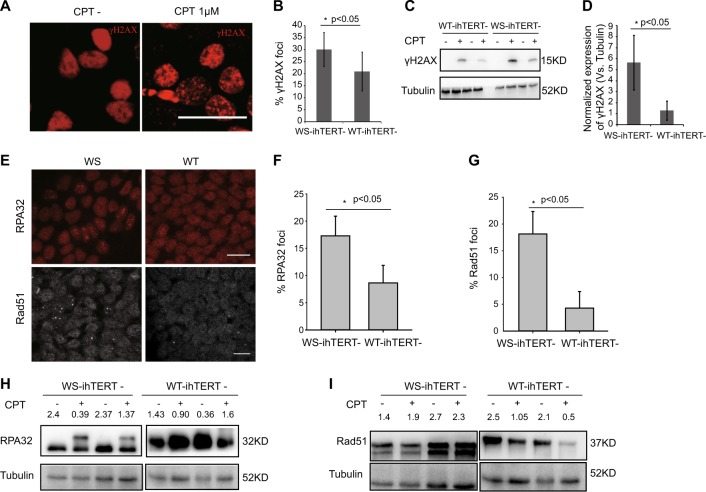
WS-iPSCs are more sensitive to the treatment of anticancer drug Camptothecin. **a** γH_2_AX staining with or without CPT treatment. **b** Percentages of γH_2_AX + foci in WS and WT groups after treatment with 1 μM CPT. **c** Western blotting analysis for γH2AX expression with or without CPT treatment. **d** Quantitative analysis of **c** (with CPT treatment). **e** Representative images of RPA32 and Rad51 immunostaining after treatment with 1 μM CPT. **f** Percentages of cells with RPA32 + foci in WS and WT groups after treatment with CPT. **g** Percentages of cells with Rad51 + foci in WS and WT groups after treatment with CPT. **h** Western blot analysis for RPA32 protein expression. **i** Western blot analysis for Rad51 protein expression. Bar = 25 μm; WS-ihTERT-, AG03141 Werner syndrome iPSCs with inducible hTERT without Dox treatment; WT-ihTERT-, AG10803 wild-type iPSCs with inducible hTERT without Dox treatment

The DSBs induced by a high dose of CPT may account for the S phase arrest in WS-iPSCs. We thus stained the cells for the formation of γH2AX foci, a DSB marker. High doses of CPT treatment induced γH2AX foci in iPSCs (Fig. 6a), and a higher percentage of CPT-treated AG03141 WS-iPSCs had γH2AX foci than did CPT-treated AG10803 WT-iPSCs (Fig. 6b). Western blotting analysis showed similar results (Fig. 6c, d). γH2AX expression in AG03141 WS cells (5.6 normalized to tubulin) was higher than in AG10803 WT ones (1.28 normalized to tubulin).

We also examined RPA32 and RAD51, two single-stranded DNA (ssDNA)-binding proteins involved in recombinational mechanisms at DSBs and stalled forks^18^. In the AG10803 WT-ihTERT (Dox-) iPSCs after treatment with CPT, RPA32, and Rad51 assumed mostly a diffusive pattern of distribution (Fig. 6e); in contrast, RPA32 and Rad51 showed an increased level of translocation to distinctive foci after CPT treatment in WS-ihTERT (Dox-) iPSCs (Fig. 6e–g). The RPA32 antibody recognizes the endogenous level of total RPA32, which did not differ in WS- vs. WT-ihTERT (Dox-) iPSCs. However, after CPT treatment, western blot showed two separate bands of RPA32 in AG03141 WS-iPSCs but not in AG10803 WT-iPSCs (Fig. 6h), and the reason for this phenomenon is yet unclear. Previous reports showed that cleavage of Rad51 to a 31-kDa protein occurs in cells undergoing apoptosis^15,19,20^. Two bands of Rad51 were detected in AG03141 WS-iPSCs but not in AG10803 WT-iPSCs (Fig. 6i).

## Discussion

Successful reprogramming of certain WS fibroblast lines were previously reported by two groups^8,9^. In Cheung’s study, five WS patient fibroblasts lines (AG03141, AG00780, AG06300, AG05229, and AG12797) were attempted for conversion to iPSCs. Among the five lines, AG03141 failed to be reprogrammed in Cheung’s study^9^. We also tested AG03141, AG00780, and AG06300. Consistent with Cheung’s report, AG03141 failed to be reprogrammed by using the Yamanaka factors, even under hypoxic conditions. The reprogramming efficiency seems to be affected by the particular genotype of WRN mutation. In our study, WS fibroblasts of homozygous truncation mutations (AG03141 and AG00780) either failed to be reprogrammed or resulted in markedly reduced efficiency of iPSC induction (Fig. 1c). Similar results were obtained using homozygous WRN knockout ESC-derived MSCs (Fig. 1d). In Shimamoto’s study, two WS patient fibroblast lines were reprogrammed to iPSCs^8^. These two WS patient cells used in Shimamoto’s study were both of compound heterozygous WRN mutation (mut4/mut6). Mut6 was a truncation mutation and mut4 was a missense mutation. WRN protein is both a helicase and an exonuclease, and contains multiple functional domains that may account for various cellular roles such as recombination, transcription, translation, repair, chromosome segregation, and telomere maintenance^1–4,13^. WRN protein with missense point mutation may still keep certain functions, which may underlie the higher reprogramming efficiency vs. cells of homozygous truncation mutations that may have little remaining or a complete loss of any residual functionality. AG03141 and AG00780 fibroblast lines were both of homozygous truncation mutations. In spite of the low induction efficiency, AG00780 could give rise to iPSCs but AG03141 could not, suggesting that other factors may also chip in and collectively determine whether the cells can be reprogrammed. AG03141 patient died of WS in her 30s, and AG00780 in his 60s. The earlier onset and death might be indicative of a more severe disease phenotype, which may be attributable to additional genetic and epigenetic factors. Indeed, senescence seems to be one of such factors. MSCs of late passage presented with signs of senescence and yielded a much lower reprogramming efficiency (Supplementary Fig. S2). The above results indicate that WS cells derived from certain patients are resistant to the conventional protocol-mediated iPSC formation, and alternative strategies are required to achieve reprograming of these particular lines.

It has been well characterized that hTERT induction and telomerase activation is required for cellular reprogramming^14,21^. Cells with telomerase deficiency due to TERT or TERC mutations display a low ability or even loss of reprogramming potential^14,22^. To elucidate the mechanism underlying reprogramming failure of AG03141 WS fibroblasts, we determined hTERT expression and telomerase activity in those cells and identified the impaired induction of endogenous hTERT expression as a key defect present in WS cells during reprogramming. Following Yamanaka factor-encoded viral infection, AG03141 WS cells exhibited limited increase in hTERT expression and telomerase activity, while its robust induction was observed in reprogrammed WT fibroblasts. Conceivably, such low levels of hTERT expression are insufficient to prevent telomere shortening, thereby inhibiting reprograming. In support of this, ectopic expression of hTERT indeed enables WS cells to undergo reprogramming, and moreover, the iPSCs derived from AG03141 WS-hTERT cells are fully pluripotent, express pluripotent markers and can differentiate into three germ layer cells both in vivo and in vitro, which were comparable to those WT iPSCs.

In addition to the defective hTERT expression observed in AG03141 WS cells, accelerated telomere shortening is likely another layer of barriers to iPSCs. The RecQ helicase is required for telomere maintenance and its loss-of-function mutation significantly impairs telomere stabilization^11,12,23^. Indeed, telomere over-erosion is widely seen in WS cells and acts as a driving-force for the onset of premature aging and aging-related disorders in WS patients^11,12,23^. Therefore, telomere dysfunction may readily happen to various degrees in WS-derived cells, and contribute to perturbation of reprograming. However, AG03141 WS and AG10803 WT fibroblasts used in the present study had comparable mean telomere length (Fig. 3i), yet iPSCs were only generated from normal WT cells. To solve this puzzle, we further performed Q-FISH at single cell levels and noticed a significant heterogeneity in TL in both WS and WT fibroblasts. Nevertheless, the fraction of AG10803 WT fibroblasts carrying longest telomere was much higher compared to that of AG03141 WS cells. Likely, a threshold level of TL is required for reprogramming to iPSCs, and cells are more advantaged with longer telomere. Consistently, hTERT was shown to robustly increase iPSC efficiency in normal cells by lengthening telomere, as shown in the present study and other reports^24^.

To determine whether constitutive expression of exogenous hTERT is required for AG03141 WS-iPSCs, we switched off its ectopic expression after cellular reprogramming by using an inducible system. AG03141 WS-iPSCs exhibited lower attachment efficiency and longer population doubling coupled with decreased BrdU incorporation upon hTERT repression. The cell cycle analysis revealed an S-phase defect associated with AG03141 WS-iPSCs. Thus, the proliferation of WS-iPSCs was apparently impaired when ectopic hTERT expression was switched off. A slow growth rate and S-phase defect was previously observed in WS-cells^25^. Likely, hTERT over-expression masked this defect via its telomere lengthening-independent mechanism.

The defect in cell cycle progression and hypersensitivity to Topoisomerase I inhibition observed in WS-derived iPSCs and WRN-/- ESCs closely mimic the intrinsic disease phenotype, which might serve as a suitable disease model for mechanistic studies.

## Materials and methods

### Generation and characterization of iPSCs

Werner syndrome fibroblasts (AG03141, AG00780, and AG06300) and wild-type control fibroblasts (AG10803) were obtained from Coriell biorepository. AG03141 fibroblasts were derived from a WS patient homozygous for a C to T mutation at nucleotide 2476 in the *WRN* gene (2476C > T), resulting in a stop codon at 748 [Gln748TER (Q748X)]. AG00780 donor subject was homozygous for a C > T transition at nucleotide 1336 in exon 9 of the *RECQL2* gene (1336C > T) resulting in an amino acid change at codon 368 from arginine to a stop codon [Arg368Ter (R368X)]. AG06300 fibroblast cell line contained the following polymorphism: a leucine for phenylalanine replacement at amino acid 1074 of the WRN protein [Phe1074Leu (F1074L)]. The protein was wild-type. Before reprogramming, human TERT was introduced to WS (AG03141) and wild-type (AG10803) fibroblasts using retroviral vectors expressing hTERT and puromycin resistant gene. After puromycin selection, fibroblast cells, which stably expressed hTERT were used to generate iPSCs. To induce iPSCs with inducible hTERT, lentiviral vectors- pLVX-Tight-Puro (Cat#:632162, Clontech. Mountain View, CA, USA,) expressing hTERT and pLVX-Tet-on advanced (Cat#:632162, Clontech) were introduced into AG03141 WS and AG10803 WT fibroblasts. Cells expressing inducible hTERT were used to generate iPSCs by using Sendai virus expressing human OCT4, SOX2, KLF4, and c-MYC. Five days after infection, cells were replated on MEF feeders in human embryonic stem cell (hES) medium containing Knock-out DMEM (KO Dulbecco’s modified Eagle’s medium), 20% KSR, 1% NEAA, 1% GlutaMAX, 0.1 mM beta-mercaptoethnol and 10 ng/ml bFGF (All from Thermo Fisher Scientific, Waltham, MA, USA). Two weeks after replating, iPSCs colonies appeared in the culture system. Colonies were picked up manually and transferred onto Martigel-coated 24-well plates and cultured in mTesR medium (Stemcell, Vancover, BC, Canada) for expansion. For passaging, confluent iPSCs were treated with Accutase at 37 °C for 2 min, washed with phosphate-buffered saline (PBS), and scraped off from the plate with the tip of a 2 ml pipette. The cell clusters were then collected into centrifuge tubes, spun down, and split in a ratio of 1:6.

To characterize the iPS clones, we tested expression of pluripotent markers Oct4, Sox2, TRA-1-81, and SSEA4 using immnocytochemical staining. iPSCs were injected into the hind limb muscle of SCID/Beige mice to test the capacity to develop teratoma. The expression of pluripotent genes OCT4, SOX2, NANOG, REX-1, ESG1, and DPPA4 were tested using RT-PCR. Karyotyping was performed and analyzed using traditional methods. Teratoma was dissected and analyzed by immunohistochemical staining.

### Embryonic stem cell culture

Human H9 embryonic stem cells and WRN-deficient hESCs were maintained on mitomycin C-inactivated mouse embryonic fibroblasts (MEFs) in human ESC medium or on Matrigel (BD Biosciences) in mTeSR medium (STEMCELL Technology). All hMSCs were cultured in hMSC culture medium (αMEM + GlutaMAX (Invitrogen), 10% fetal bovine serum (FBS, Gemcell), 1% penicillin/streptomycin (Invitrogen), 0.1 mM non-essential amino acids (Gibco) and 1 ng/ml bFGF (Joint Protein Central)).

### CRISPR/Cas9-mediated WRN knockout in H9 ES cells

Two homozygous WRN knockout ES Cells were used in the study. WRN-ES1 line has been published previously^13^. A new homozygous knockout ESC line WRN-ES2 was generated for this study. CRISPR/Cas9-mediated WRN editing in hESC was performed as previously described^26^. In brief, WRN gRNA (TACATAAACAGGTGGATAC) targeting Exon 14 of WRN was cloned into gRNA-mCherry vector (WRN-gRNA-mCherry). H9 ESCs were treated with ROCK inhibitor Y-27632 (TOCRIS) for 24 h. The next day, individualized hESCs (5 × 10^6^) were resuspended in 100 μl opti-MEM (Gibco) containing 12 μg Cas9-GFP and 8 μg WRN-gRNA-mCherry and were then electroporated by 4D-Nucleofector (Lonza). After electroporation, cells were seeded on Matrigel-coated plates in mTeSR. After 48 h expansion, dual-positive cells were collected by FACS machine (BD FACS Aria II) and plated on MEF feeder in human ESC medium. Emerging clones were manually picked into 96-well plates and expanded on Matrigel-coated plates for genotyping and western blotting.

### Generation and characterization of hMSCs

Differentiation of H9 and WRN-deficient hMSCs from hESCs were performed as previously described^27^. Briefly, hESCs were dissociated into EBs and then were plated on Matrigel-coated plates in differentiation medium (αMEM + GlutaMAX (Invitrogen), 10% FBS (Gemcell), 10 ng/ml of bFGF (Joint Protein Central), 5 ng/ml of TGFβ (HumanZyme) and 1% penicillin/streptomycin) for around 10 days. The confluent MSC-like cells were passaged on gelatin-coated plates and then were sorted by FACS to purify CD73/CD90/CD105 triple-positive hMSCs.

### Alkaline phosphatase assay

Alkaline phosphatase (AP) staining was performed using BCIP/NBT Alkaline Phosphatase Color development kit (Beyotime Biotechnology, Wuhan, China) according to the manufacturer-provided protocol. Briefly, reprogrammed fibroblasts and MSCs on Day 21 were fixed with 4% PFA for 10 min at room temperature (RT). Then cells were washed three times for 5 min/time with PBS. AP staining solution was freshly prepared by mixing solution BCIP and NBT with staining buffer. For one well of a 6-well-plate, 1 ml staining solution was added and incubated for 30 min at RT in the dark. Reaction was stopped by replacing staining buffer with distilled water. Then cells were ready for image capturing.

### Teratoma assay

For teratoma analysis, 5 × 10^6^ hES cells suspending in Matrigel/mTeSR (1:4) were injected subcutaneously into NOD-SCID mice (6–8 weeks). Animals were sacrificed at around 8 weeks to collect teratoma. Teratoma were fixed in 4% paraformaldehyde, dehydrated in 30% sucrose, embedded in O.C.T. compound (TISSUE-TEK), for sectioned while frozen, and analyzed by immunostaining for the expression of the lineage markers of endoderm (anti-FOXA2), mesoderm (anti-SMA) and ectoderm (anti-TUJ1).

### Telomere quantitative FISH (Q-FISH) and flow-FISH analysis

Telomere Q-FISH on iPSCs were performed according to the method published before^28^. Briefly, iPSCs collected at different passages were spun onto Superfrost plus slides. The slides were subjected to freeze-thaw cycles in liquid nitrogen, and incubated in 0.1 N HCL for 10 min. The PNA-telomere probe (PANAGENE Inc., Daejeon, Korea) was then added. High-resolution images were collected and analyzed using Leica Confocal TCS SP5 with 488 nm sequential laser scan.

Flow-FISH on iPSCs was performed according to a method published before with minor modifications^29^. Briefly, iPSCs were first fixed with 4% PFA for 10 min at RT. After 10 min wash in phosphate-buffered saline with Tween 20 (PBST), cells were resuspended in PBS with 20% glycerol and incubated for 45 min at RT. Then cell pellets collected from the previous step were frozen and thawed for 3–5 cycles in liquid nitrogen. Cell membrane was removed through the treatment. Treated cells were ready for telomere probe hybridization after three times rinse (each time with PBST for 5 min, 0.1 N HCl for 5 min, and PBST for 5 min). For hybridization, cells were first mixed thoroughly with 170 μl hybridization mixture in PCR tubes and incubated for exactly 10 min at RT in the dark. Tubes with hybridization mixture were transferred to a mental rack in 87 °C water bath and incubated for 15 min. Tubes were taken out from water bath and hybridized for 90–120 min at RT in the dark. After hybridization, cell suspension was transferred from PCR tubes to 1.5 ml eppendorf tubes prefilled with 900 μl wash buffer. Cell suspension were centrifuged for 5 min at 1500 × g at 16 °C to collect cell pellet. Supernatant was aspirated until 100 μl buffer remained and then another 1 ml wash buffer was added. This wash step was repeated four times. Cell pellets were finally resuspended in 300 μl counterstaining solution and incubated on ice for 20–60 min. Now samples were ready to be tested by flow cytometery. The experiments were run with a flow cytometer (BD callibur II) and analyzed using FlowJo.

### Telomerase activity assay

The telomerase activity was measured using Telo TAGGG Telomerase PCR ELISA kit (Roche Bioscience, Palo Alto, CA, USA). Briefly, cell pellets were treated with 200 μl lysis reagent and incubated for 30 min on ice. The lysate was centrifuged at 16,000 × *g* for 20 min at 4 °C. The supernatant was collected and aliquoted in a proper volume with new pre-cooled EP tubes. Unused supernatant was stored at -80 °C. Ten microliters supernatant were heated at 85 °C water bath as a negative control in the following PCR reaction. The PCR-based analysis was carried out in a 50 μl reaction mixture containing 2 μl cell lysate. Samples, positive controls (provided by the kit), and negative controls were amplified according to the conditions provided by manufactures. PCR product was denatured and hybridized to digoxigenine-(DIG)-labeled, telomeric repeat-specific detection probe. The resulting product was immobilized via the biotin-labeled primers to a streptavidin-coated microplate. Telomerase activity was detected by measuring the absorbance of the samples at 450 nm.

### Synchronization and cell cycle analysis

WS and WT iPSCs were plated at the same density on the first day. On the 3rd day, cells were synchronized at G2/M phase by treatment with 200 ng/ml nocodazole (Sigma-Aldrich, St. Louis, MO, USA) for 16 h. After recovery for 5 h in normal culture medium (iPSCs begin to enter S-phase at this time point), cells were collected and fixed with cold 70% ethanol every 2 h. To induce DNA replication stress in S-phase, cells were treated with 250 nM CPT (Sigma-Aldrich), followed by propidium iodide (PI) staining and FACS analysis.

### Apoptosis analysis

iPSCs were cultured in a feeder-free culture system with mTesR. Cells were separately treated with 250 nM CPT (37 °C, 5% CO_2_, 1 h), 50 μM bleomycin (37 °C, 5% CO_2_, 30 min), 1 μM brefeldin A and 500 μM H_2_O_2_ (5 min). CPT is a cytotoxic quinoline alkaloid that inhibits the DNA topoisomerase I. CPT induces replication stalling and DNA breaks in a concentration-dependent manner. Bleomycin is another anticancer drug that can also induce DNA strand breaks. Although the exact mechanisms of DNA strand scission are unresolved, it has been suggested that hydroxide free radicals may be involved. Brefeldin A inhibits protein transport from the endoplasmic reticulum to the golgi apparatus and shows cytotoxic effects in certain cancer cell lines. H_2_O_2_ is a reactive oxygen species that can readily react with and damage vital cellular processes/components, such as DNA synthesis and mitochondria functions. Treated cells were stained with Annexin V-FITC and PI according to the protocol provided by the manufacture (Annexin V-FITC/PI Apoptosis Detection Kit). Labeled cells were analyzed by using Flow Cytometer (BD, Callibur II).

### Western blot analysis

Western blot analysis was performed according to the standard protocol. Primary antibodies include anti-WRN (ab200, Abcam, Cambridge, MA, USA), γH2AX (9718, Cell Signaling Technology (CST), Danvers, MA, USA), RPA32 (2208, CST), RAD51 (sc-8349,Santa Cruz Biotechnology, Dallas, Texas, USA), and Actin (CW0096M, CWBIO, Beijing, China).

### Immunofluorescence staining

The procedure has been described in a previously published study^30^. The primary antibodies used were as follows: anti-OCT4 (1:400, 2750,CST), anti-TRA-1-60 (1:300, MAB4360, EMD Millipore, Billerica, MA, USA), anti-SSEA-4 (1:100, MAB4304, Millipore), anti-SOX2 (1:1000, MAB4343, Millipore), anti-TRA-1-81 (1:200, 14-8883, eBioscience), anti-AFP (1:200, 14-6583, eBioscience), anti-α-SMA (1:100, ab8207, Abcam), anti-Tuj-1 (1:500, MAB1637, Millipore), anti-WRN(1:200, ab200, Abcam), anti-γH2AX (1:200, 9718, CST), anti-RPA32(1:400, 2208, CST), anti-Rad 51 (1:400, sc-8349, Santa Cruz), anti-NANOG (1:400, ab80892, Abcam), and anti-FOXA2 (1:400, 3143, CST).

### Statistical analysis

All comparisons between WS and wild-type groups were tested by unpaired, two-tailed Student’s *t*-test. Comparison between more than two groups was performed using ANOVA. A *p*-value < 0.05 was considered statistically significant.

## Electronic supplementary material

Figure S1

Figure S2

Figure S3

Figure S4

Figure S5

Figure S6

Figure S7

Supplementary figure legends
