# Supplementary material for: Ectopic hTERT expression facilitates reprograming of fibroblasts derived from patients with Werner syndrome as a WS cellular model
Source: Cell Death Dis. 2018 Sep 11;9(9):923. doi: 10.1038/s41419-018-0948-4 (PMC6134116; doi:10.1038/s41419-018-0948-4)
Supplement: Supplementary file 1 — Figure S1 [file 41419_2018_948_MOESM1_ESM.pdf]

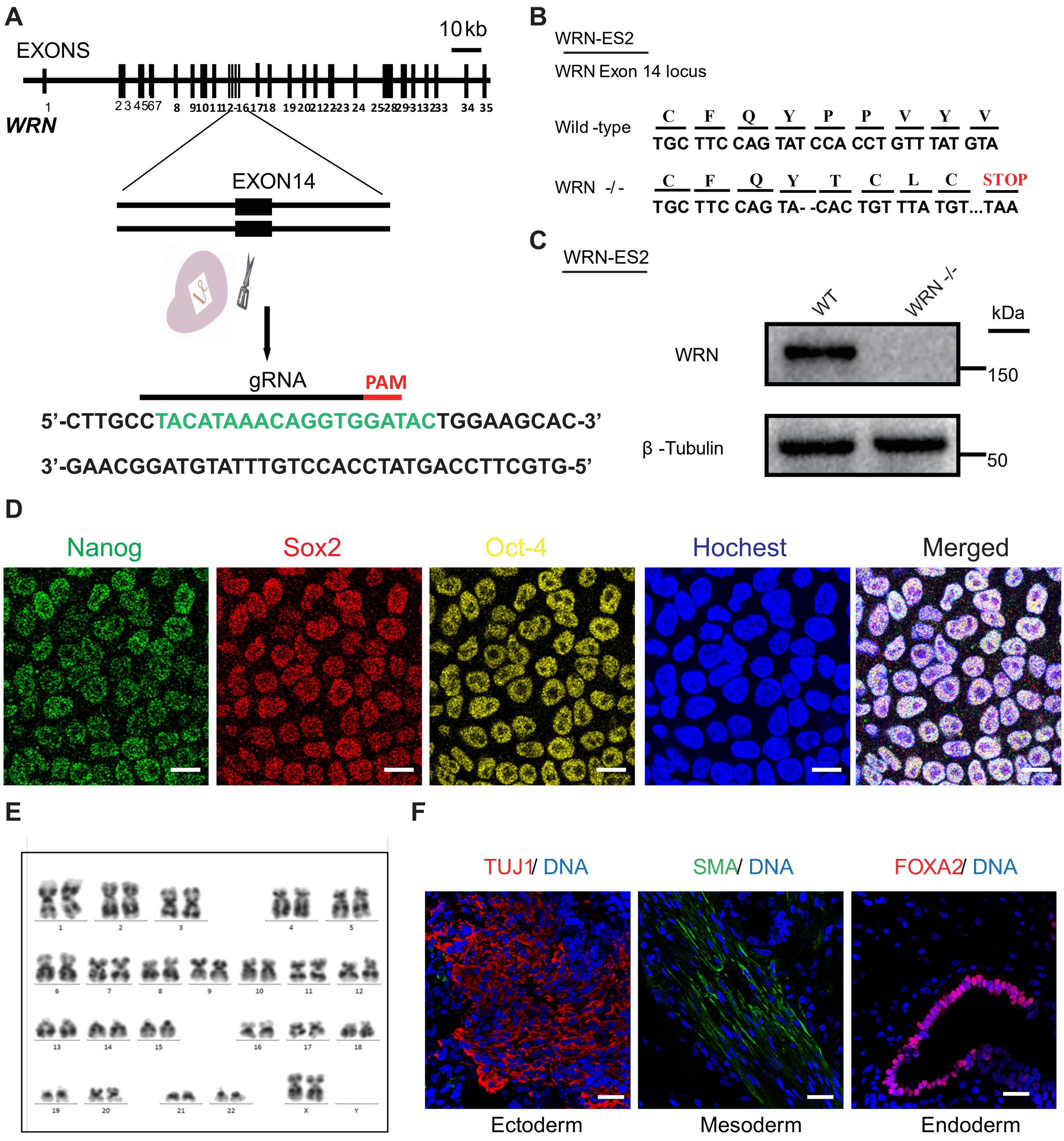

A
10 kb
EXONS
1
2 3 4 5 6 7 8 9 10 11 12-16 17 18 19 20 21 22 23 24 25 28 29 31 32 33 34 35
WRN
EXON14
gRNA
PAM
5'-CTTGCCTACATAAACAGGTGGATACTGGAAGCAC-3'
3'-GAACGGATGTATTTGTCCACCTATGACCTTCGTG-5'
B
WRN-ES2
WRN Exon 14 locus
Wild -type
C F Q Y P P V Y V
TGC TTC CAG TAT CCA CCT GTT TAT GTA
WRN -/-
C F Q Y T C L C STOP
TGC TTC CAG TA- -CAC TGT TTA TGT...TAA
C
WRN-ES2
WT
WRN -/-
kDa
WRN
150
β -Tubulin
50
D
Nanog
Sox2
Oct-4
Hochest
Merged
E
F
TUJ1/ DNA
SMA/ DNA
FOXA2/ DNA
Ectoderm
Mesoderm
Endoderm
