# Supplementary figures and images for: Ectopic hTERT expression facilitates reprograming of fibroblasts derived from patients with Werner syndrome as a WS cellular model
Source: Cell Death Dis. 2018 Sep 11;9(9):923. doi: 10.1038/s41419-018-0948-4 (PMC6134116; doi:10.1038/s41419-018-0948-4)

**A**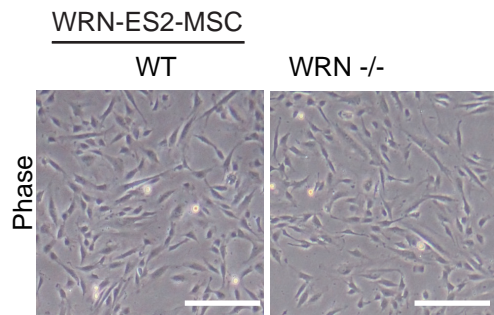**B**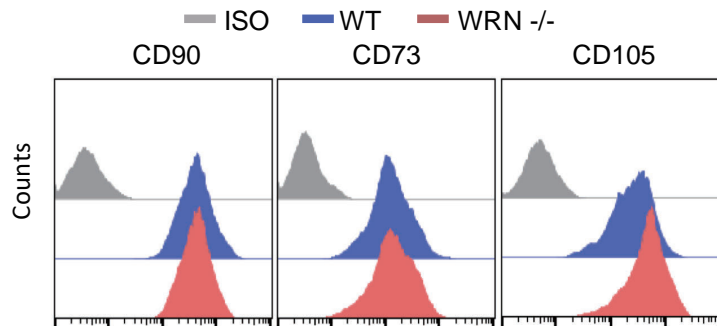**C**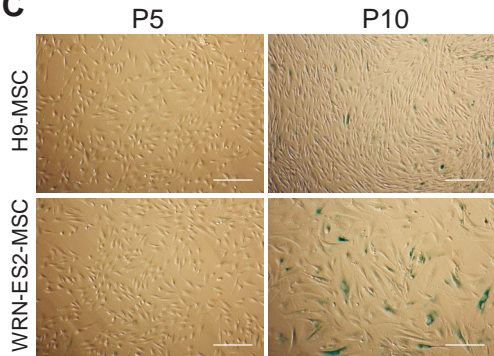**D**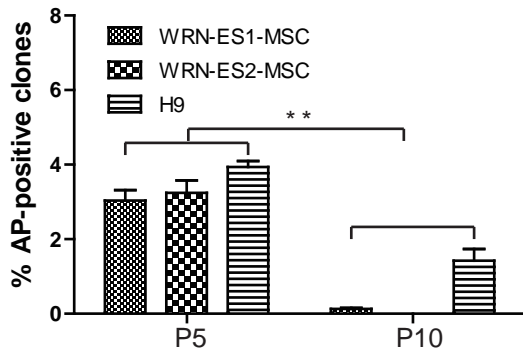

Supplement: Supplementary file 2 — Figure S2 [file 41419_2018_948_MOESM2_ESM.pdf]

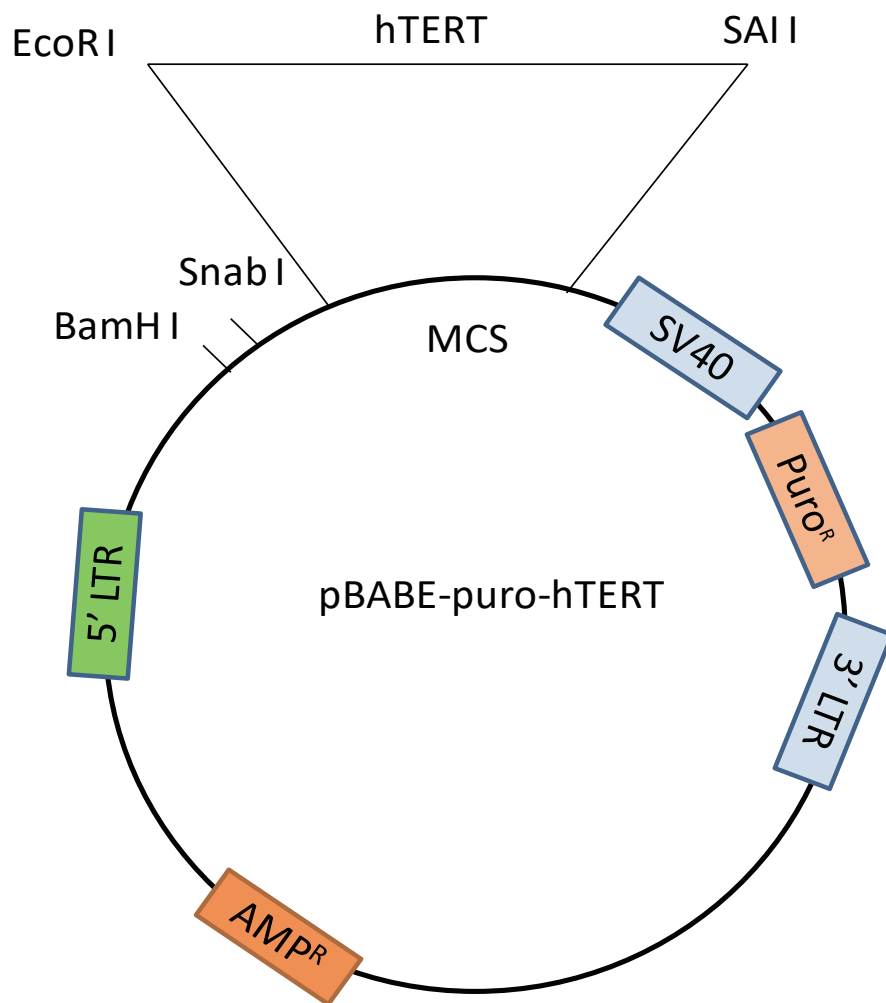

Supplement: Supplementary file 3 — Figure S3 [file 41419_2018_948_MOESM3_ESM.pdf]

**A**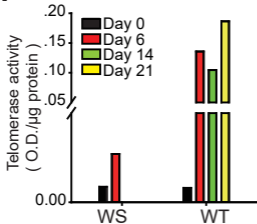**B**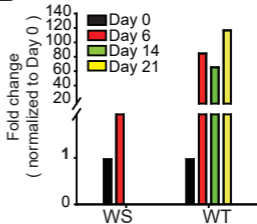

Supplement: Supplementary file 4 — Figure S4 [file 41419_2018_948_MOESM4_ESM.pdf]

A

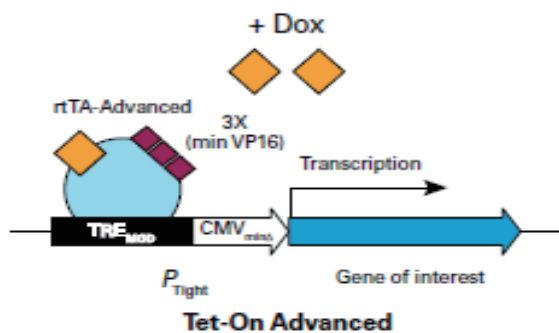

B

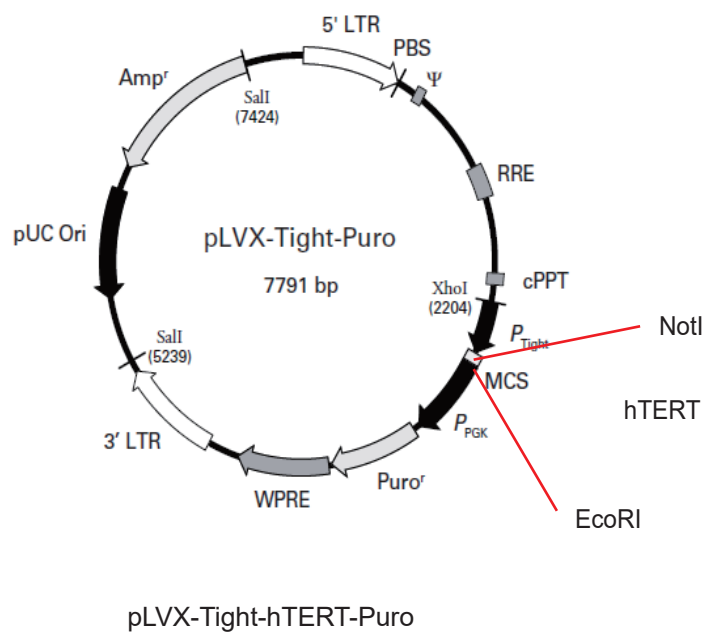

C

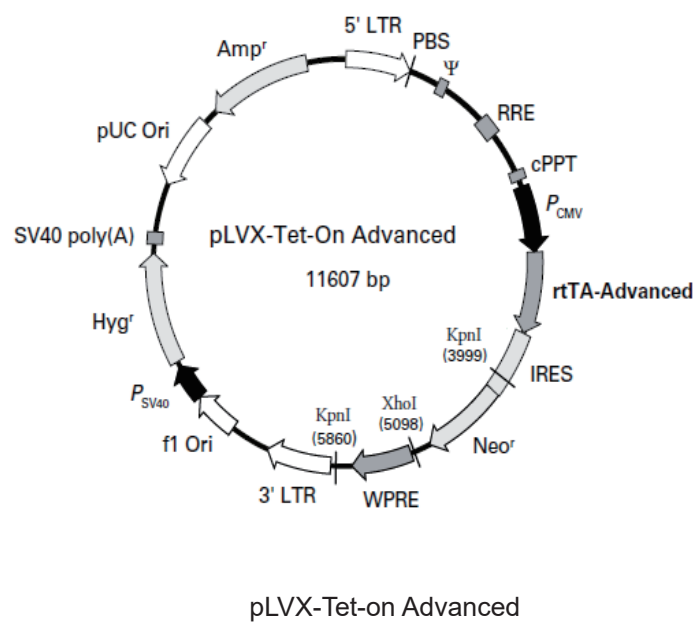

D

GFP/p-Tight

GFP / p-Tight Dox+

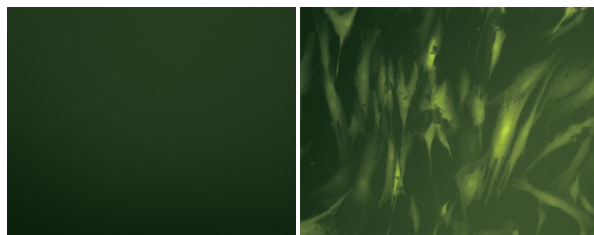

E

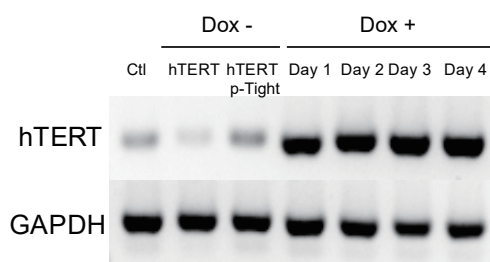

Supplement: Supplementary file 5 — Figure S5 [file 41419_2018_948_MOESM5_ESM.pdf]

**A**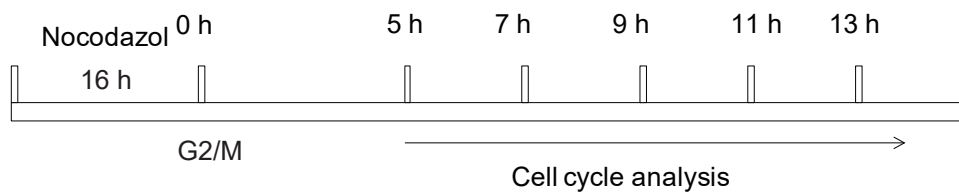**0 h** **WS-ihTERT B3 -**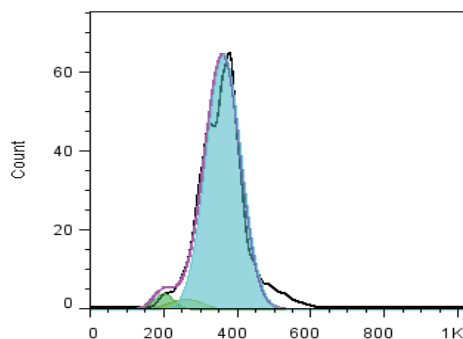**WRN-ES1**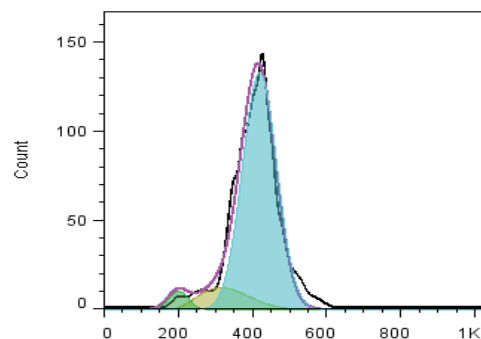**5 h** **WS-ihTERT B3 -**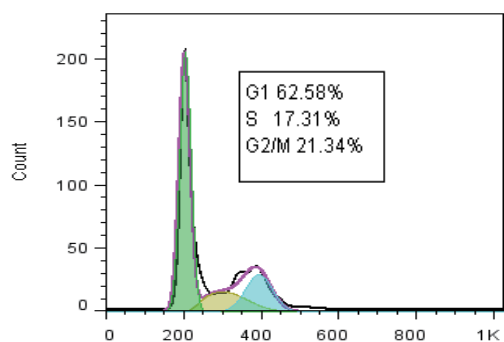**WT-ihTERT G11 -**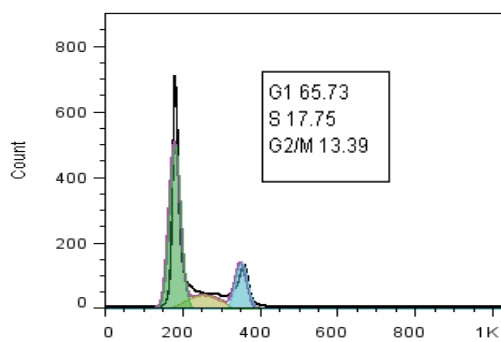**5 h** **WRN-ES1**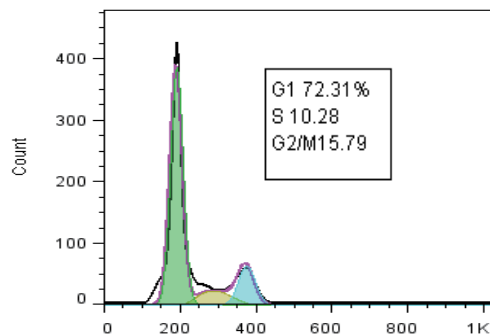**H9**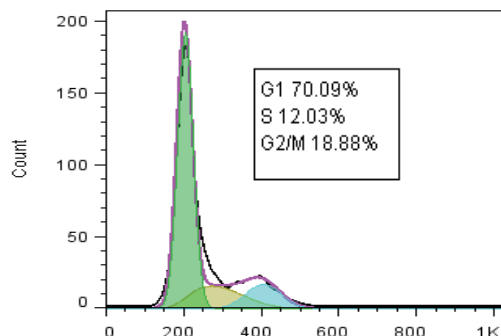

Supplement: Supplementary file 6 — Figure S6 [file 41419_2018_948_MOESM6_ESM.pdf]

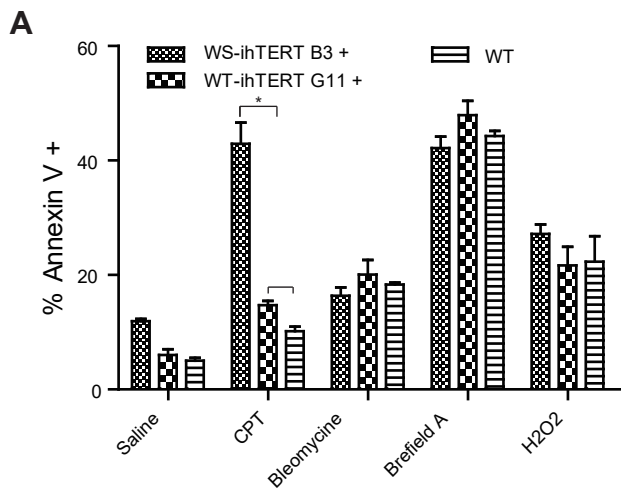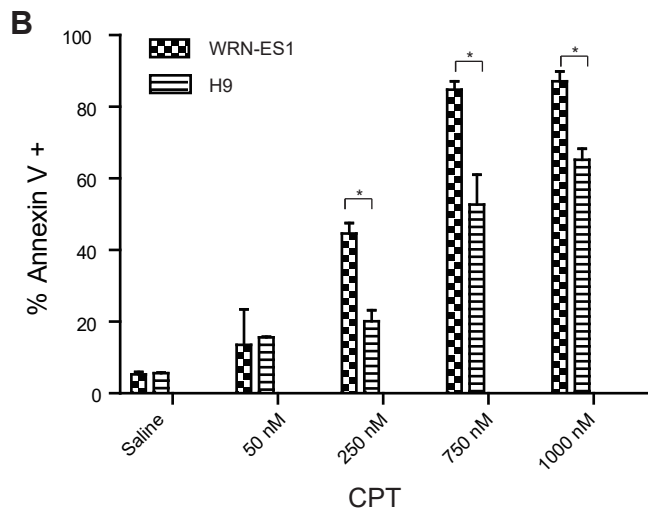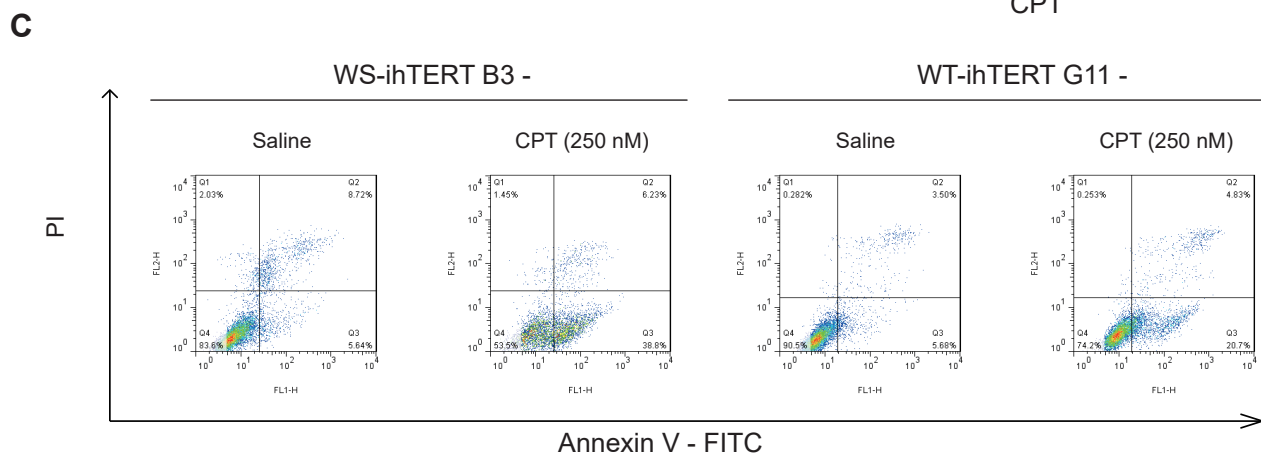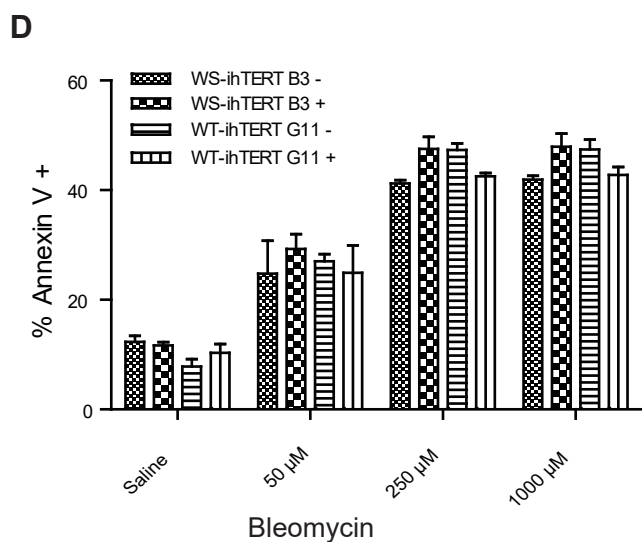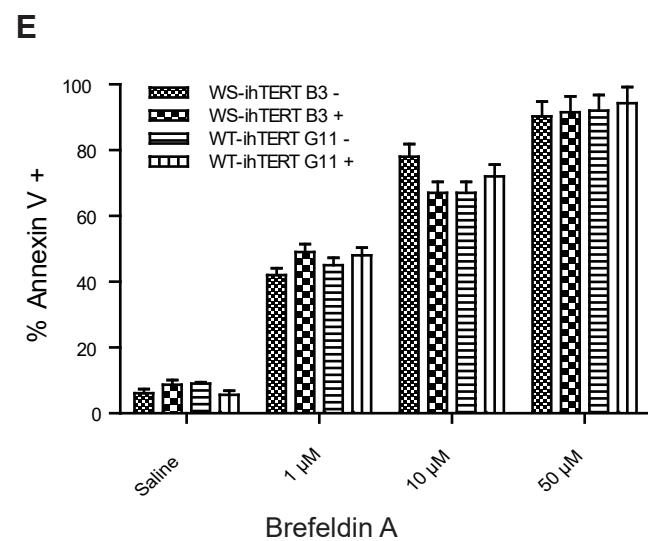

Supplement: Supplementary file 7 — Figure S7 [file 41419_2018_948_MOESM7_ESM.pdf]
