# Supplementary material for: Ectopic hTERT expression facilitates reprograming of fibroblasts derived from patients with Werner syndrome as a WS cellular model
Source: Cell Death Dis. 2018 Sep 11;9(9):923. doi: 10.1038/s41419-018-0948-4 (PMC6134116; doi:10.1038/s41419-018-0948-4)
Supplement: Supplementary file 8 — Supplementary figure legends [file 41419_2018_948_MOESM8_ESM.docx]

**Supplementary Figure Legends**

**Fig. S1.** Generation and characterization of WRN Knockout embryonic stem cells.

A, B) Genotyping analysis of CRISPR/Cas9-mediated WRN knockout hESCs.

C) Western blot analysis demonstrated the absence of WRN protein in CRISPR/Cas9-mediated WRN knockout hESCs. β-Tublin was used as a loading control.

D) Representative immunofluorescence images of CRISPR/Cas9-mediated WRN knockout hESCs showing expression of pluripotency markers. Scale bar, 20 μm

E) G-banded karyotyping analysis of CRISPR/Cas9-mediated WRN knockout hESCs showing that they had normal karyotypes.

F) Immunostaining for representative markers of the three germ layers in teratomas developed from CRISPR/Cas9-mediated WRN knockout hESCs. Scale bar, 30 μm.

**Fig. S2.** High passage number and cellular senescence may increase the barrier of MSCs to iPSC induction.

A) Representative images of H9-derived and WRN-ES2-derived MSCs.

B) FACS results of CD90, CD73 and CD105 expression on ES-derived MSCs (WRN-ES2-MSCs and H9-MSCs).

C) β-gal staining results on early (P5) and late passage (P10) MSCs derived from WRN-ES2 and H9 ES cells.

D) iPSC induction efficiencies of early (P5) and late (P10) passage MSCs differentiated from WRN-ES1, WRN-ES2 and H9 ES cells. Values represent the mean percentage of alkaline phosphatase (AP)-positive clones on Day 21 among the number of plated cells.

**Fig. S3.** pBAB3- hTERT-puro retroviral vector information.

hTERT gene was subcloned into the multiple cloning site (MCS).

**Fig. S4.** Defective telomerase activation occurs during reprograming of WS fibroblasts.

A) Telomerase activity of WS (AG03141) and WT (AG10803) groups at reprogramming Day 0, 6, 14, and 21.

B) Fold change of telomerase activity at different reprogramming time points (Day 6, Day 14 and Day 21) normalized to that of Day 0 in WS (AG03141) and WT (AG10803) groups.

**Fig. S5**. pLVX-Tight-hTERT-Puro vector information and inducibility assay results.

A) Gene induction in the Tet-On® Advanced Systems. Tet-On Advanced Systems are active in the presence of Dox.

B, C) Vector maps of pLVX-Tight-hTERT-Puro and pLVX-Tet-on Advanced. hTERT gene was subcloned between Not I and EcoR I.

D) Dox-inducible GFP expression in fibroblasts infected with pLVX-Tight-GFP-Puro and pLVX-Tet-on advanced vectors.

E) Inducible hTERT expression in iPSCs with or without Dox treatment.

Ctl, Control iPSCs without exogenous vector;

hTERT, iPSCs expressing pLVX-Tight-hTERT-Puro only;

hTERT p-Tight, iPSCs co-expressing pLVX-Tight-hTERT-Puro and pLVX-Tet-on advance.

**Fig. S6.** Nocodazol treatment synchronizes iPSCs/ESCs at G2/M phase.

A) Scheme of cell cycle synchronization.

B) Cell cycle analysis of WS iPSCs, WRN-ES cells, and respective wild-type cells at 0 h and 5 h.

**Fig. S7.** WS-iPSCs are more sensitive to the treatment of anticancer drug Camptothecin.

A) Percentages of apoptotic iPSCs treated with Camptothecin (CPT), Bleomycin, Brefeldin A, or H_2_O_2._

B) Percentages of apoptotic (Annexin V +) ESCs after treatment with different concentrations of CPT. * P<0.005

C) Annexin V flow cytometry results of WS- and WT-iPSCs under inducible TERT expression off status (Dox -) with or without CTP treatment.

D) Percentages of apoptotic (Annexin V +) iPSCs after treatment with different concentrations of Bleomycin.

E) Percentages of apoptotic (Annexin V +) iPSCs after treatment with different concentrations of Brefeldin A.
